# Novel Magnetic Resonance Imaging-Based Method for Accurate Diagnosis of Meniere's Disease

**DOI:** 10.3389/fsurg.2021.671624

**Published:** 2021-06-22

**Authors:** Taeko Ito, Takashi Inoue, Hiroshi Inui, Toshiteru Miyasaka, Toshiaki Yamanaka, Kimihiko Kichikawa, Noriaki Takeda, Masato Kasahara, Tadashi Kitahara, Shinji Naganawa

**Affiliations:** ^1^Department of Otolaryngology-Head and Neck Surgery, Nara Medical University, Kashihara, Japan; ^2^Institute for Clinical and Translational Science, Nara Medical University, Kashihara, Japan; ^3^Inui ENT Clinic, Sakurai, Japan; ^4^Department of Radiology, Nara Medical University, Kashihara, Japan; ^5^Department of Otolaryngology, University of Tokushima Graduate School of Medicine, Tokushima, Japan; ^6^Department of Radiology, Nagoya University Graduate School of Medicine, Nagoya, Japan

**Keywords:** Meniere's disease, magnetic resonance imaging, endolymphatic hydrops, diagnostic strategy, ROC analysis

## Abstract

**Background:** Pathologically, Meniere's disease symptoms are considered to be associated with endolymphatic hydrops. Examinations revealing endolymphatic hydrops can be useful for accurate Meniere's disease diagnosis. We previously reported a quantitative method for evaluating endolymphatic hydrops, i.e., by measuring the volume of the endolymphatic space using three-dimensional magnetic resonance imaging (MRI) of the inner ear. This study aimed to confirm the usefulness of our methods for diagnosing Meniere's disease. Here, we extracted new explanatory factors for diagnosing Meniere's disease by comparing the volume of the endolymphatic space between healthy volunteers and patients with Meniere's disease. Additionally, we validated our method by comparing its diagnostic accuracy with that of the conventional method.

**Methods and Findings:** This is a prospective diagnostic accuracy study performed at vertigo/dizziness centre of our university hospital, a tertiary hospital. Eighty-six patients with definite unilateral Meniere's disease and 47 healthy volunteers (25 and 33 males, and 22 and 53 females in the control and patient groups, respectively) were enrolled. All participants underwent 3-Tesla MRI 4 h after intravenous injection of gadolinium to reveal the endolymphatic space. The volume of the endolymphatic space was measured and a model for Meniere's disease diagnosis was constructed and compared with models using conventional criteria to confirm the effectiveness of the methods used. The area under the receiver operating characteristic curve of the method proposed in this study was excellent (0.924), and significantly higher than that derived using the conventional criteria (0.877). The four indices, sensitivity, specificity, positive predictive value, and negative predictive value, were given at the threshold; all of these indices achieved higher scores for the 3D model compared to the 2D model. Cross-validation of the models revealed that the improvement was due to the incorporation of the semi-circular canals.

**Conclusions:** Our method showed high diagnostic accuracy for Meniere's disease. Additionally, we revealed the importance of observing the semi-circular canals for Meniere's disease diagnosis. The proposed method can contribute toward providing effective symptomatic relief in Meniere's disease.

## Introduction

Meniere's disease (MD) is a common disease characterized by recurrent vertigo/dizziness and cochlear symptoms (hearing loss, tinnitus, and aural fullness). Since a well-constructed clinical guideline for MD has already been reported (1) patients with MD can receive appropriate treatment once the correct diagnosis has been reached. In 2015, new criteria for MD diagnosis were established by a collaborative working group led by the Barany Society (2). In the Barany criteria, MD diagnosis is mainly based on patients' subjective symptoms. Therefore, for accurate diagnosis and appropriate treatment, obtaining an accurate medical history and clarification of patients' complaints are essential. However, this can be challenging, since patient complains are often vague. In fact, 40–80% of patients are not definitively diagnosed in the long term, thus leading to inadequate treatment, (3) administration of unnecessary medicine, and increases in medical costs, which are significant problems worldwide. Thus, examinations that help reach an accurate diagnosis with minimal requirements for specialist training are desirable.

In 1938, Yamakawa (4) and Hallpike and Cairns (5) independently reported the finding of endolymphatic hydrops in the temporal bones of patients with MD; hence, objective examinations that can reveal endolymphatic hydrops are considered to be useful for diagnosing MD, and many investigators have attempted to establish them. Electrocochleography and glycerol and furosemide testing are used for diagnosing endolymphatic hydrops, but the positivity rate of these tests among patients with MD is ~50% (6–12). Because of the low ratios, these tests cannot be components of the diagnostic criteria of MD, and it is considered difficult to clinically detect endolymphatic hydrops in patients with MD by morphological examinations.

In 2006, the Nagoya University group succeeded in visualizing the endolymphatic space using 3-Tesla MRI after intravenous gadolinium injection (13) (inner ear MRI [ieMRI]). Subsequently, a convenient method of grading endolymphatic hydrops on ieMRI was proposed (14). Using this grading, the positive ratio of endolymphatic hydrops on ieMRI was found to be 70–100% (15–17) suggesting that it could be a diagnostic criterion of MD. Although this grading has the advantage of being convenient, it has the limitation of being a qualitative evaluation, using only one slice of ieMRI, with the plane for image analysis varying depending on the head position and anatomical differences in the skull, and restricted to a particular part of the inner ear, neglecting the semi-circular canals (SCCs). To allow ieMRI to be a component of the diagnostic criteria of MD, it is required to establish a quantitative method to measure endolymphatic hydrops in the whole inner ear and identify the explanatory factors for their grading; hence, we started to measure the volumes of inner ears and endolymphatic space in 2016 (18–20). In these previous studies, to quantitatively observe endolymphatic hydrops in the whole inner ear, we reported a method of constructing high-quality three-dimensional images of ieMRI (ie3DMRI) and the volume of the endolymphatic space.

In the present study, we compared the volume of the endolymphatic space between patients with MD and healthy controls using the ie3DMRI method, thereby extracting new and reliable explanatory factors for diagnosing MD. These factors can help clinicians to diagnose MD with accuracy.

## Methods

The Medical Ethics Committee of our university approved this study (certificate number: 0889). Written informed consent was obtained from each participant, including healthy volunteers.

### Patients and Controls

We enrolled 86 patients diagnosed with unilateral Meniere's disease (uMD), according to the Barany criteria (2) and American Academy of Otolaryngology-Head and Neck Surgery (21) at the vertigo/dizziness center of our university hospital between July 2014 and December 2018. As controls, 47 healthy volunteers participated in the present study, and data related to both ears (*n* = 94) of each volunteer were included in the statistical analysis. Control volunteers had no history of hearing loss, vertigo, middle or inner ear diseases, cranial disease, head trauma, renal disease, or heart disease. The controls were not using any medications at the time. Both patients and controls had no history of allergy to gadolinium.

### MRI Observation

MRI measurements were acquired 4 h after intravenous administration of a single dose (0.1 mmol/kg body weight) of gadolinium-diethylenetriaminepentaacetic acid dimethylamide (Magnescope; Guerbet, Tokyo, Japan) (15). A 3-Tesla MRI unit (MAGNETOM Verio; Siemens, Erlangen, Germany) with a 32-channel array head coil was used. The sequences, proposed by Naganawa et al. (22) specific to endolymphatic and perilymphatic fluids were adopted. A radiologist (with 14 years of experience) and two otolaryngologists (with 12 and 29 years of experience), who were blinded to the clinical progress of the patients, evaluated the MRI findings independently. If their evaluations differed, a third otolaryngologist (with 31 years of experience) made the final decision.

### Conventional Two-Dimensional Evaluation of Endolymphatic Hydrops

The criteria proposed by Nakashima et al. (14) and Kahn et al. (23) were adopted. According to the criteria by Naganawa et al. we classified the endolymphatic hydrops in the cochleae and vestibules as none, mild, or significant. In the present study, both mild and significant were defined as positive: the existence of endolymphatic hydrops. Additionally, we evaluated the endolymphatic hydrops using the criteria by Kahn et al. and classified them as none, grade I, or grade II; grade I, or grade II was defined as positive in the present study.

### Volumetric Measurement of Total Fluid Space and Endolymphatic Space

Volumetric measurement of the total fluid space and endolymphatic space was performed in accordance with our previously published methods (18–20) on our workstation (Virtual Place; AZE, Ltd., Tokyo, Japan). In this protocol, the endolymphatic space voxels have negative signal values and the perilymph space voxels have positive signal values. The volume of the total fluid space was acquired using a software to count voxels automatically. Then, the ratio of the volume of the endolymphatic space to that of the total fluid space was calculated (“ELS ratio”). We performed these measurements three times, and the average was used for calculation. In the present study, we evaluated the SCCs in addition to the cochlea and vestibule, which is the conventional method used.

### Statistical Methods

All P-values were two sided, and P-values of 0·05 or lower were considered statistically significant. All statistical analyses were performed using R, Version 3.5.2 (24). The minimum number of subjects was determined from the number of explanatory variables in logistic regression analysis; *n* = 70 for patients with uMD and *n* = 35 for controls. We examined sex and age differences between patients and controls using Fisher's exact test and the Mann–Whitney U test, respectively. The ELS ratio between controls and patients with uMD was compared using the Mann–Whitney U test. The association between patient background and the morbidity of uMD was investigated using multivariate logistic regression analysis. A model to diagnose uMD was developed on the basis of the receiver operating characteristic (ROC) curve using multivariate logistic regression analyses, and the difference in the areas under the curve (AUCs) was statistically compared using Delong's test. Moreover, we evaluated the index of net reclassification improvement (NRI) (25) and performed cross-validation among models.

## Results

### Patient Background

There were 25 and 33 men and 22 and 53 women in the control and patient groups, respectively. The mean age of controls and patients with uMD was 58.4 ± 16.3 and 56.9 ± 14.7 years, respectively. There were no significant differences between the controls and patients with uMD (sex; *p* = 0.11, Fisher's exact test, age; *p* = 0.47, Mann–Whitney U test).

### ELS Ratio of Controls and Patients With uMD

**Figure 1:**
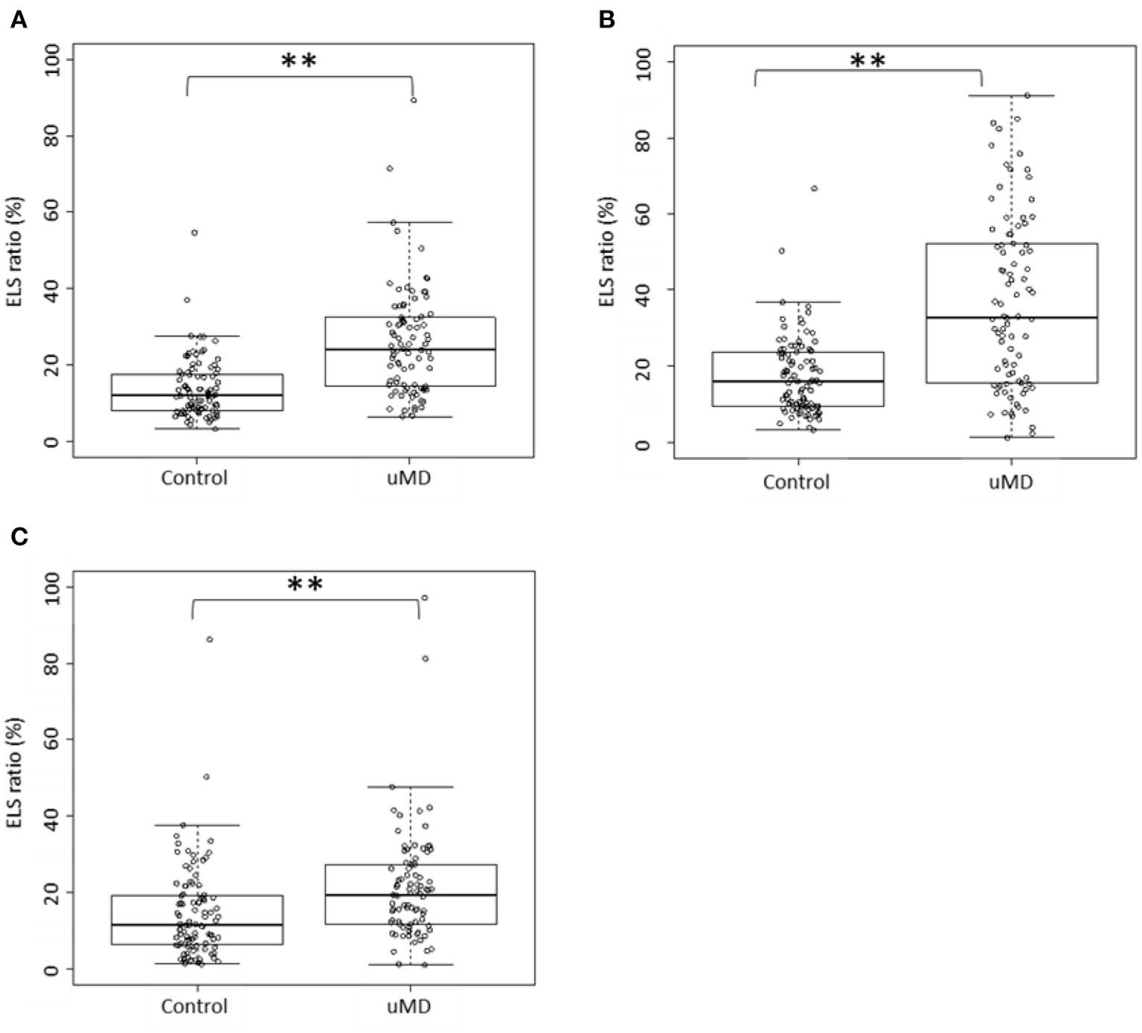
Distribution of the ELS ratio. **(A)**: cochlea; **(B)**: vestibule; **(C)**: SCCs. In the cochleae, the ELS ratios of controls and patients with uMD were 8.15% (median) and 22.50%, respectively **(A)**. In the vestibules, the ELS ratios of controls and patients with uMD were 16.25 and 32.85%, respectively **(B)**. In the SCCs, the ELS ratios of controls and patients with uMD were 11.65 and 19.50%, respectively **(C)**. The ELS ratios of patients with uMD were significantly higher than those of controls in the cochleae, vestibules, and SCCs. **p < 0.001, Mann–Whitney U test.

The Box and bee swarm plots in Figures 1A–C show the ELS ratio of controls and patients with uMD. In the cochleae, the ELS ratio of controls was 8.15 [4.93–14.45]% (median [interquartile range]) and that of patients with uMD was 22.50 [12.20–31.12]% (Figure 1A). In the vestibules, the ELS ratio of controls was 16.25 [9.70–23.65]% and that of patients with uMD was 32.85 [15.75–52.20]% (Figure 1B). In the SCCs, the ELS ratio of controls was 11.65 [6.45–19.25]% and that of patients with uMD was 19.50 [11.90–27.15]% (Figure 1C). Among all parts of the inner ears, there were significant differences between controls and patients with uMD (*p* < 0.001, Mann–Whitney U test).

### Relationship Between the ELS Ratio and the Interval From Onset of MD to MRI Observation

**Figure 2:**
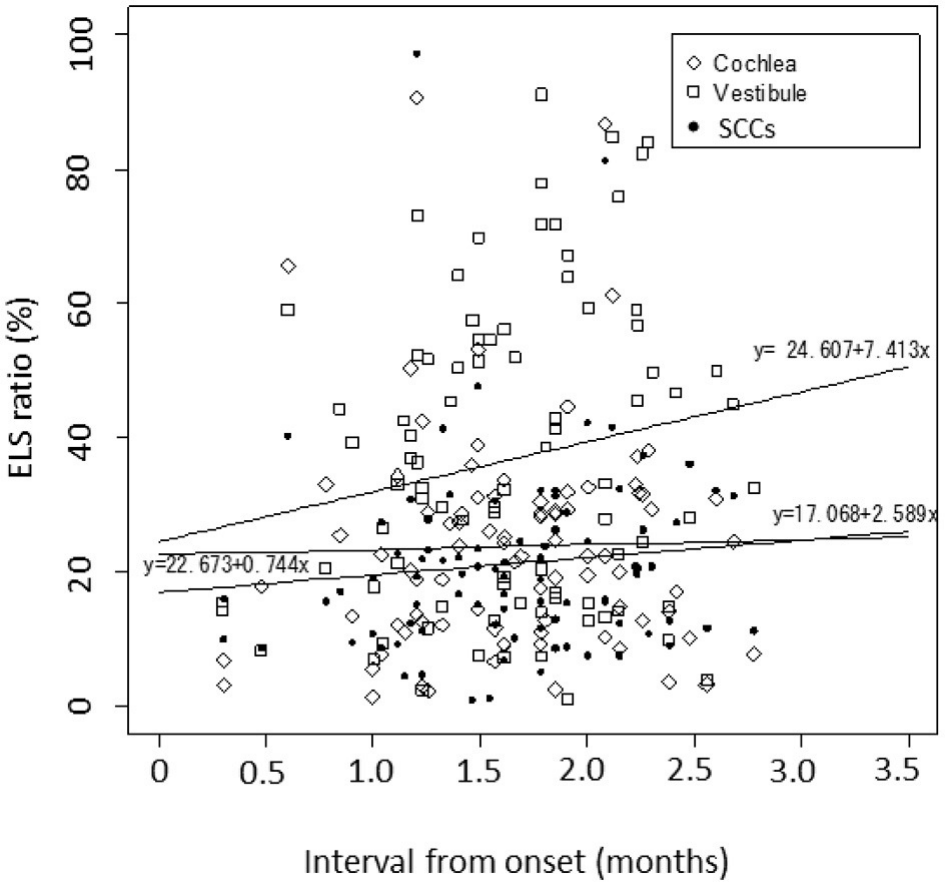
Correlation between the interval from the onset of uMD to MRI observation. Using an interval of days on a linear scale, the logarithmic day (x) is scaled as x = log_10_(1 + interval). The simple linear regression analyses summarize the data as follows: the ELS ratios (y) showed a slightly increasing trend with time, but there were no significant changes; y = 22.673 + 0.744x with *P* = 0.83 for the cochleae, y = 24·607 + 7·413x with *p* = 0.11 for the vestibules, and y = 17.068 + 2.589x with *p* = 0.38 for the SCCs. Diamond: cochlea, square: vestibule, dot: SCCs.

Figure 2 shows the correlation between the interval from the onset of uMD to MRI observation and the ELS ratio in the cochleae/vestibules/SCCs. Using an interval of days on a linear scale, the logarithmic day (x) was scaled as x = log_10_(1 + interval). The ELS ratios (y) showed a slightly increasing trend with time, but there were no significant changes (*p* = 0.83 in the cochleae, *p* = 0.11 in the vestibules, and *p* = 0.38 in the SCCs).

### Contribution of the Background Factors

Multivariate logistic regression analyses were performed with uMD as an objective variable and sex, age, and the volumes of endolymphatic hydrops in the cochleae/vestibules/SCCs as explanatory variables (Supplementary Table 1). The odds ratio of being female was significantly higher in patients with uMD; sex was a confounding factor for a causality pathway toward MD and should be included as an adjustment factor in model building for diagnosing uMD. The ELS ratio in SCCs was not a significant factor in this analysis.

### Model Building for MD Diagnosis

Logistic regression models were built for diagnosing MD. We introduced an abridged notation to describe logistic regression equations, abbreviating the regression coefficients and expressing the first-order interaction between predictors *A*_1_ and *A*_2_ as *A*_1_:*A*_2_. Consider *Y*~*A*_1_ + *A*_2_ + *A*_1_:*A*_2_ as an example; this notation denotes *logit*{P/(1-P)} = β_0_ + β_1_*A*_1_ + β_2_*A*_2_ + β_c_*A*_1_·*A*_2_. Here, the objective and dichotomous variable *Y* is predicted by the probability P at which *Y* gains 1 and β represents a regression coefficient. Each regression coefficient for each predictor is determined using the maximum likelihood estimation based on a set of predictors for given observational data.

The results obtained from the conventional evaluation of endolymphatic hydrops on ieMRI (15) were applied to a logistic regression model to diagnose MD. An objective and dichotomous variable is *MD*, determined according to the Barany criteria (2). Predictors were *Sex, EHV*, and *EHC*, where *EHV* and *EHC* were the morbidity indicators of endolymphatic hydrops in the vestibule and cochlea, respectively. Values of these variables were given as 0, 0.5, or 1 for no, mild, or significant endolymphatic hydrops, respectively, according to the conventional grading (14). The objective variable *MD* was predicted with Equation (1) [2D model], where the interaction term *EHV:EHC* was omitted because trial calculations showed that it was not correlated with the objective variable *MD*:

(1) $$\begin{array}{l} MD\sim Sex+EHV+EHC \end{array}$$

We built a logistic regression model from a set of volumes of vestibules, cochleae, or SCCs and their ELS, evaluated using the ie3DMRI technique. Seven independent predictors were used: *Sex* (Sex), *Vv* (vestibular volume), *Cv* (cochlear volume), *Sv* (SCC volume), *V*h (ELS volume in the vestibule), *Ch* (ELS volume in the cochlea), and *Sh* (ELS volume in the SCC). Each of other predictors was defined as a function of the seven independent predictors and selected from the standpoint of medicine: *V*i = 1/*Vv, V*r = *V*h·*V*i, *V*r*2* = *V*r^2^, *C*i = 1/*Cv, C*r = *C*h·*C*i, *C*r*2* = *C*r^2^, *S*i = 1/*Sv, S*r = *S*h·*S*i, *S*r*2* = *S*r^2^, *VC*i = 1/(*Vv* + *Cv*), *VC*r = (*V*h + *C*h)·*VC*i, *VC*r*2* = *VC*r^2^, *I*i = 1/(*Vv* + *Cv* + *Sv*), *I*r = (*V*h + *C*h + *S*h)·*I*i, *I*r*2* = *I*r^2^.

To improve the MD diagnostic accuracy, we selected predictors in the logistic regression model to maximize the AUC of the ROC curve and to minimize Akaike's information criterion (25) (AIC) using the data from 94 ears of controls and 86 ears of patients with uMD. The model validity was confirmed using a cross-validation method of “leave-one-out calculation.” The result is presented using Equation (2) [3D model] (Supplementary Table 2).

(2) $$\begin{array}{l} MD\sim Sex+Vv+Cv+Sv+Vi+Si+VCi+Vh+Vr+Cr+\\ \;VCr+Ir+Vr2+Cr2+Vr:Cr+Sr:VCr+VCr:Ir \end{array}$$

### Comparison of uMD Diagnostic Accuracy of Models

**Figure 3:**
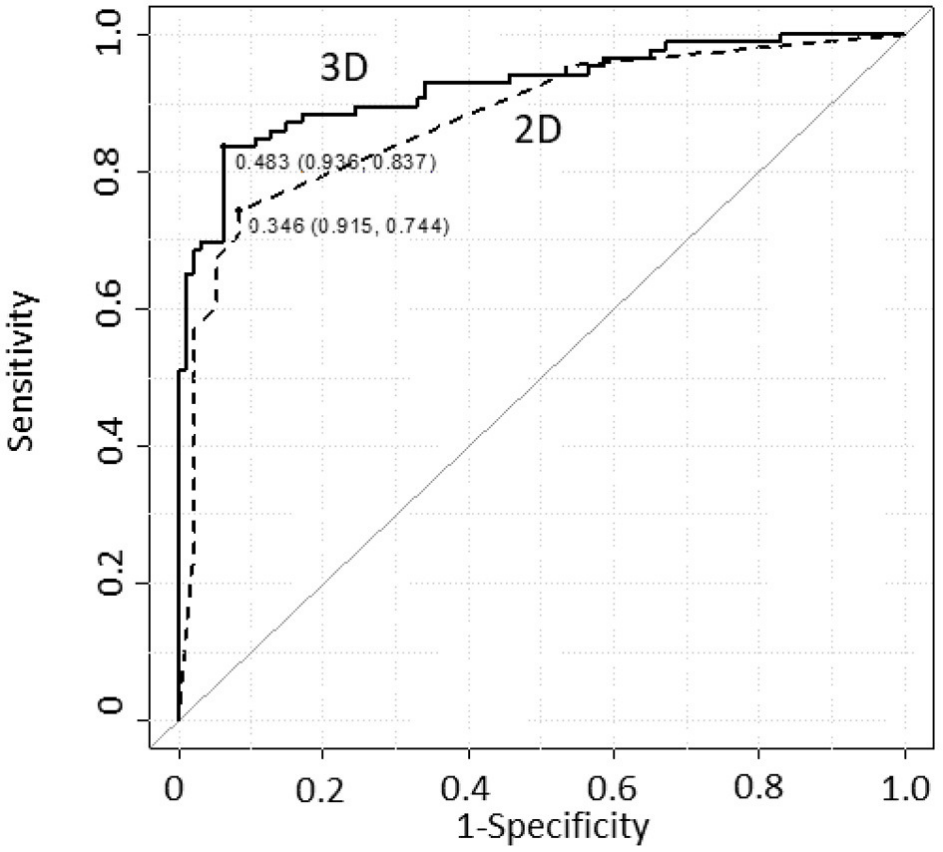
ROC curve comparison for MD diagnostic accuracy between the models. The AIC values were 161.3 and 155.7 with the 2D and 3D models, respectively. The AUC values were 0.877 and 0.924 with the 2D and 3D models, respectively. For the 3D model, both AIC and AUC values were improved compared with those for the 2D model. 2D Model: MD~Sex + EHV + EHC. 3D Model: MD~Sex + Vv + Cv + Sv + Vi + Si + VCi + Vh + Vr + Cr + VCr + Ir + Vr2 + Cr2 + Vr:Cr + Sr:VCr + VCr:Ir. Dotted line: 2D model, continuous line: 3D model.

We conducted MD diagnosis on 86 ears of patients with uMD and 94 ears of controls and compared the diagnostic accuracy between the two models. Figure 3 shows ROC curves and thresholds for MD diagnosis, with a solid line plotted according to the logistic regression model, based on Equation (2) [3D model], and a dotted line according to the conventional method, (21) based on Equation (1) [2D model] for reference.

The MD diagnostic accuracies of the two models were tabulated and compared (Table 1). Each threshold value was determined as the closest top-left point on the ROC curve. The four indices, i.e., sensitivity, specificity, positive predictive value, and negative predictive value, were given at the threshold; all of these indices achieved higher scores for the 3D model. The AIC value improved from 161.3 to 155.7 (2D−3D model), and the AUC value improved from 0.877 to 0.924 (2D−3D model); however, Delong's test showed that the AUC difference was not significant.

**Table 1:** Comparison of MD diagnostic accuracy between the models.

**Subjects: uMD +control**	**Threshold**	**Sensitivity**	**Specificity**	**Positive predictive value**	**Negative predictive value**	**AUC**	***p*-value**	***p*-value by NRI**	**AIC**
2D model	0.346	0.744	0.915	0.889	0.796	0.877			161.3
3D model	0.483	0.837	0.936	0.923	0.863	0.924	*p* = 0.07	*p* <0.001	155.7

*Each threshold value was determined as the closest top-left point on the ROC curve. The four indices of sensitivity, specificity, positive predictive value, and negative predictive value were given at the threshold; all of these indices achieved higher scores for the 3D model. The AIC value improved from 161.3 to 155.7 (2D−3D model), and the AUC value improved from 0.877 to 0.924 (2D−3D model)*.

**Figure 4:**
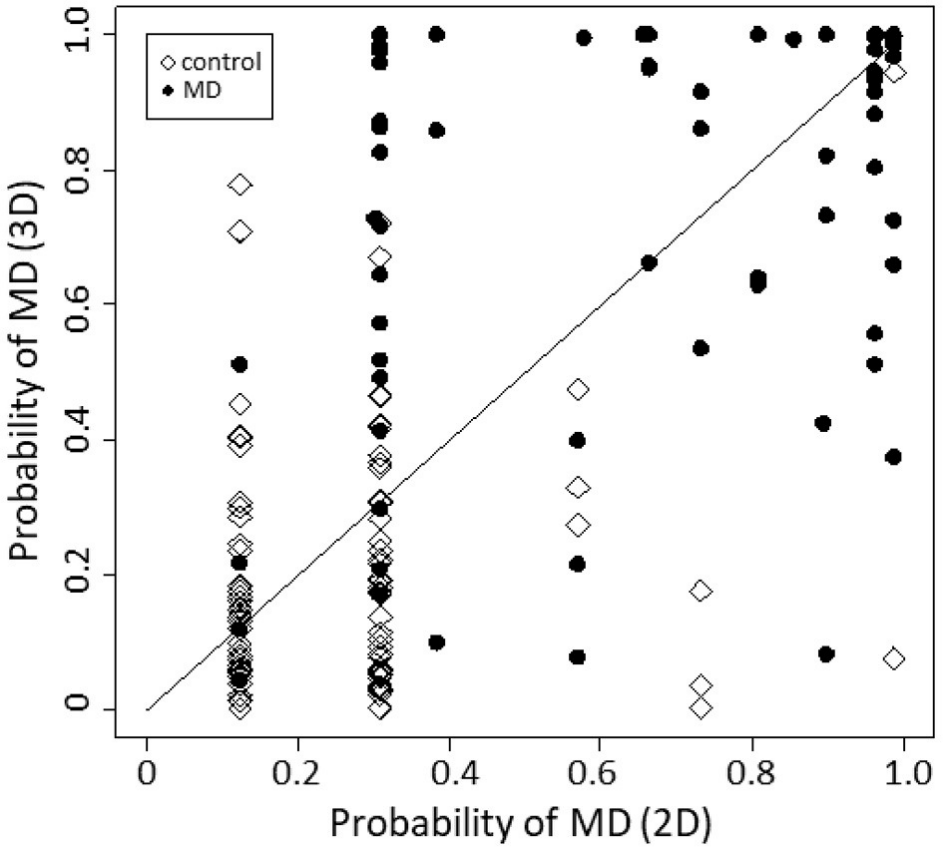
Reclassification plot, a basis for the NRI test. The diagnostic accuracy was significantly improved by the 3D model compared with the 2D model (*p* < 0.001, continuous NRI test).

Figure 4 is a reclassification plot showing the diagnostic improvement with the 3D model compared with the 2D model. Based on the evaluation, the following continuous NRI test was carried out, and the improvement in diagnostic accuracy was statistically confirmed (*p* < 0.001).

A cross-validation procedure (26) was conducted (Supplementary Table 3), in which the AICs, AUCs, and mean square errors were calculated for the models' varying explanatory terms. The mean square error was evaluated using the leave-one-out method as the mean square of the difference between the estimated MD probability and the definite diagnosis for the remaining subjects. The selected 3D model minimized the AIC and mean square error, implying that improvement in MD diagnostic accuracy of the selected 3D model from the 2D model was achieved by the incorporation of terms related to SCCs and the interaction between the cochleae, vestibules, and SCCs.

### Sensitivity and Specificity of the Selected 3D Model and the 2D Model

The sensitivity of the selected 3D model and the 2D model was 0.83 (71/86) and 0.74 (64/86), respectively. The specificity of the selected 3D model and the 2D model was 0.94 (88/94) and 0.93 (87/94), respectively.

## Discussion

In the present study, we confirmed that our method using ie3DMRI strongly supported the diagnosis of MD with a high level of accuracy. Additionally, when we optimized the MD diagnostic formulae, incorporating the ELS ratio of the SCCs and the interaction among the whole inner ear, cochleae, vestibules, and SCCs, the ROC curves significantly improved. Cross-validation of the models showed that the improvement was due to the incorporation of both SCCs and the interaction between the cochleae and vestibules, although the ELS ratio in the SCCs was not a significant factor for MD. This implies that the ELS ratio in the SCCs and its interaction with the cochleae and vestibules are associated with morbidity in MD. Moreover, we succeeded in incorporating the interaction between the ELS ratio of the cochleae, vestibules, and SCCs in the MD diagnosis, whereas the conventional grading could evaluate endolymphatic hydrops only in the cochlea and vestibule. In fact, some patients with intractable MD had endolymphatic hydrops in the SCCs; in our previous study, symptomatic relief was associated with the disappearance of endolymphatic hydrops in the SCCs after endolymphatic sac drainage (20). Sugimoto et al. also reported that some patients showed endolymphatic hydrops herniation into the SCCs (27). Taken together, we should not neglect the endolymphatic space in SCCs while diagnosing MD and elucidating the causes of MD symptoms.

Wu et al. and Gürkov et al. reported that endolymphatic hydrops showed progressive deterioration during the disease duration from the data of 41 (28) and 54 patients, (29) respectively. In the present study (*n* = 86), although the ELS ratio tended to increase with time, no significant changes were noted. Based on the results in the present study, our method has the potential to distinguish individuals with MD from those without, even in the early stages of disease progression.

One of the limitations of this study was that symptoms were not included in our analysis. The Barany criteria, which are presently the most reliable criteria, emphasize the importance of interpreting the characteristics of vertigo/dizziness in diagnosing MD (2). Inclusion of data related to the characteristics of vertigo/dizziness in the statistical analysis may have led to more accurate results. In the present study, however, we decided to exclude these factors to evaluate the usefulness of ie3DMRI *per se*. A high rate of accurate diagnosis can be reached when combining the method in the present study with the conventional strategy for MD diagnosis. Misdiagnoses of vertigo/dizziness especially by non-specialists are major concerns, resulting in increased medical costs (3, 30). The method in the present study could also provide valuable feedback to non-specialists and residents who wish to polish their vertigo diagnostic skills. Another limitation was that not all patients with MD had endolymphatic hydrops and not all patients with endolymphatic hydrops had MD. The results indicated a factor other than endolymphatic hydrops caused the MD symptoms in some cases. The relationship between MD symptoms and endolymphatic hydrops remains unclear. When the causes of MD symptoms are revealed, they should be included to the factors entered in MD diagnostic models, which can lead to improved MD diagnosis accuracy and symptom relief for a larger number of patients.

For a long time, researchers studying MD have attempted to elucidate the mechanisms of endolymphatic hydrops formation and the relationship between symptoms and changes in the condition of endolymphatic hydrops. Volumetric measurement of ELS, which is objective, and quantitative observation of endolymphatic hydrops in patients has the potential to elucidate these issues in the future.

## Data Availability Statement

The original contributions presented in the study are included in the article/Supplementary Material, further inquiries can be directed to the corresponding author/s.

## Ethics Statement

The studies involving human participants were reviewed and approved by The Medical Ethics Committee of Nara Medical University. The patients/participants provided their written informed consent to participate in this study.

## Author Contributions

TIt and TIn contributed equally to the manuscript. TIt, TIn, and TK have full access to all of the data in the present study and take responsibility for the integrity of the data and the accuracy of the data analysis. TIt, TIn, TY, KK, NT, MK, TK, and SN were involved in the study conception and design. TIn provided statistical expertise. TIt and TIn drafted the manuscript. TIt, TK, and SN are the guarantors. All the authors participated in the interpretation of the results and critical revision of the manuscript.

## Conflict of Interest

The authors declare that the research was conducted in the absence of any commercial or financial relationships that could be construed as a potential conflict of interest.
