# Supplementary material for: Novel Magnetic Resonance Imaging-Based Method for Accurate Diagnosis of Meniere's Disease
Source: Front Surg. 2021 Jun 22;8:671624. doi: 10.3389/fsurg.2021.671624 (PMC8257926; doi:10.3389/fsurg.2021.671624)
Supplement: Supplementary file 1 [file Data_Sheet_1.pdf]

Supplementary material

Figure 1: Reconstruction procedure for color rendering and fusion of three-dimensional images of the inner ear fluid space and the endolymphatic space (ELS).

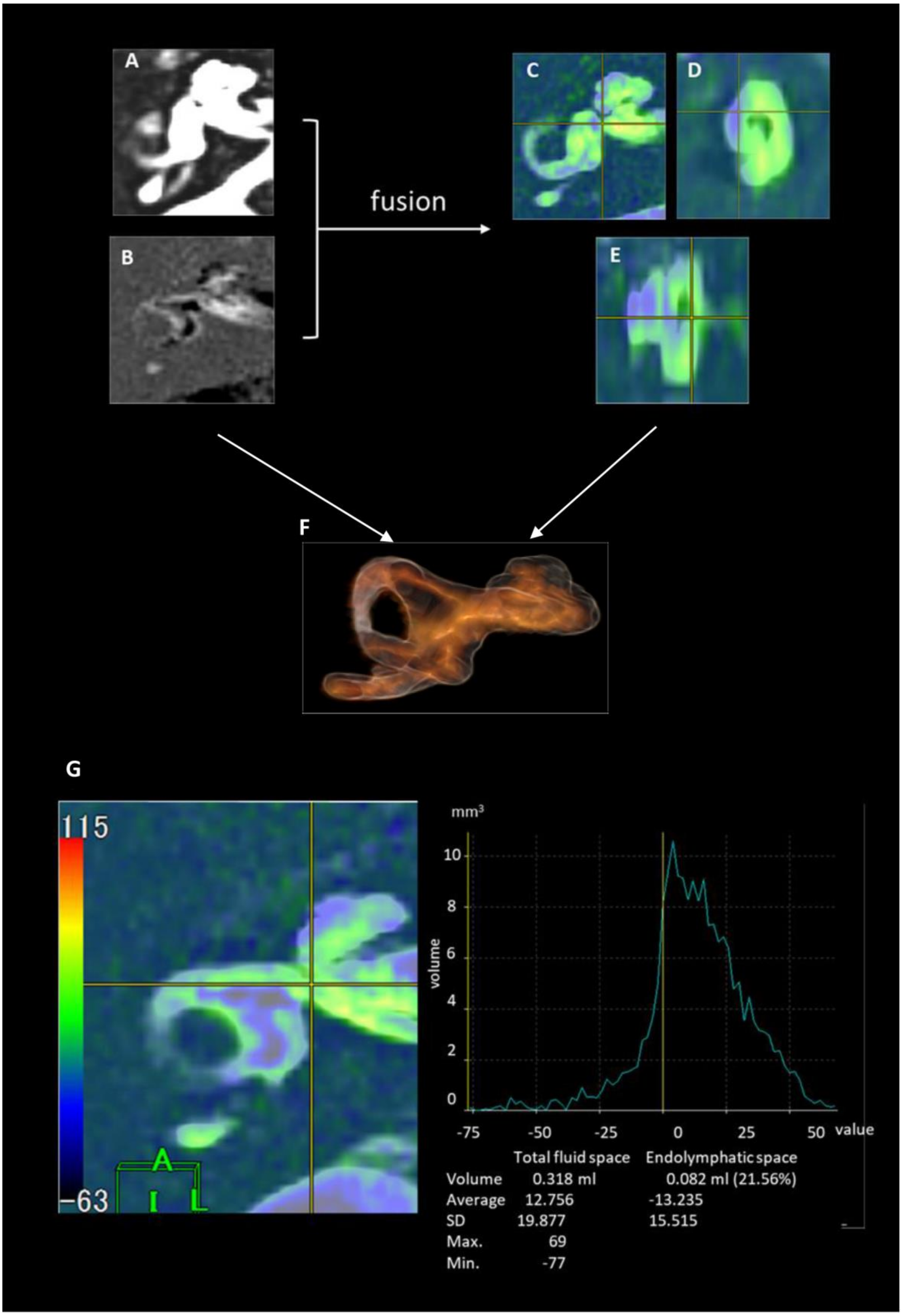

(A) Image of the total inner ear fluid space; (B) image of the ELS; (C) fusion images: axial view of the inner ear; (D) fusion images: sagittal view of the inner ear; (E) fusion images: coronal view of the inner ear; (F) a model of the three dimensional constructed images of the inner ear MRI, (G) a graph used for the measurement of the volume of the total inner ear fluid space and ELS.

Heavily T2-weighted (hT2W) MR cisternography was used for anatomical total lymph fluid reference. The hT2W 3D fluid-attenuated inversion recovery sequences with an inversion time of 2250 ms yielded positive perilymph images (PPIs), and hT2W 3D inversion recovery with an inversion time of 2050 ms yielded positive endolymph images (PEIs). A hybrid of the reversed image of the positive endolymph signal and the negative image of the positive perilymph signal was then obtained by subtracting PEI from PPI, as proposed by Naganawa et al. The image of the total inner ear fluid space obtained using the T2-SPACE sequence was fused with the image of the ELS by subtracting the positive endolymph image from the positive perilymph image (PPI-PEI). The cochlea, vestibule, and three ampullae of the semi-circular canals (SCCs) were identified on the SPACE sequence image in our workstation.[1] Our workstation automatically constructed the three dimensional models of the inner ear (F); the white lines, the transparent areas and the orange areas indicated the bonny inner ear, the perilymphatic space and the endolymphatic space, respectively. The volume of the total fluid space was acquired using software to automatically count voxels. The volume of the ELS was measured by counting the voxels of the negative signals on the ELS image.
The grey area on the color bar (G) indicates a value of 0 on the graph (yellow line); a negative value indicates the volume of the ELS.

Table 1: Multivariate logistic regression analysis on morbidity of MD

		Multivariate logistic regression analysis	
		**Odds ratio (95% CI) for uMD**	**p-value of coefficient**
Sex (Male)		0.449 (0.213, 0.943)	p = 0.04
Age (years)		0.996 (0.973, 1.020)	p = 0.76
ELS ratio (%)	Cochlea	1.43×10^4 (103, 199E6)	p < 0.001
	Vestibule	78.9 (3.38, 1.84×10^3)	p = 0.007
	SCCs	0.17 (0.005, 6.13)	p =0.33

The odds ratios of sex and age were 0.449 (p = 0.04) and 0.996 (p = 0.76), respectively. The odds ratio of factor of female to that of male was significantly higher in patients with uMD. Among the background factors, sex should be included as an adjustment factor in model building for the diagnosis of uMD. The odds ratios of the ELS ratio in the cochlea, vestibule, and SCCs were 1.43×10^4 (p<0.001), 78.9 (p = 0.007), and 0.167 (p = 0.33), respectively. The ELS ratios in the cochleae and vestibules were significant factors, but the ELS ratio in the SCCs was not a significant factor in the analysis for constructing diagnostic models for MD.

Table 2: Coefficients of the selected 3D model

	Estimated coeff.	S.E.	z value	p-value
(Intercept)	-7.73	27.18	-0.28	0.78
Sex	-1.09	0.54	-2.01	0.04 *
Vv	476.6	152.6	3.12	0.002 **
Cv	-143.9	83.82	-1.72	0.09 .
Sv	-109.1	69.22	-1.58	0.11
Vi	2.58	0.77	3.38	0.001 ***
Si	-1.5	0.75	-1.93	0.05 ·
VCi	-4.13	2.46	-1.68	0.09 ·
Vh	-421.1	239.1	-1.76	0.08 ·
Vr	-163.7	41.96	-3.90	p< 0.001 ***
Cr	-272.8	69.03	-3.95	p< 0.001 ***
VCr	391.3	101.8	3.84	p< 0.001 ***
Ir	74.47	18.95	3.93	p< 0.001 ***
Vr2	206.4	47.65	4.33	p< 0.001 ***
Cr2	419.9	97.19	4.32	p< 0.001 ***
Vr:Cr	403.7	106.3	3.80	p< 0.001 ***
VCr:Sr	325.1	90.88	3.58	p< 0.001 ***
VCr:Ir	-1319.2	319.8	-4.13	p< 0.001 ***

S.E.: standard error
coeff: coefficient
***: p< 0.001, **: p<0.01, *: p<0.05, .: p<0.1.

Regression coefficients of the selected 3D model were determined using the maximum likelihood estimation, based on a set of predictors for given observational data.

Table 3: Cross-validation for model selection

Model No.	Model 2D/3D	Incorporation			Performance		
		SCCs	Interaction terms	No. of explanatory terms	AUC	AIC	Mean square error
0	Conventional 2D model	No	No	3	0.877	161.3	0.14
1	3D model	No	No	10	0.856	187.8	0.172
2	3D model	No	Yes	11	0.858	180.5	0.164
3	3D model	Yes	No	14	0.871	181.9	0.172
4	The selected 3D model	Yes	Yes	17	0.924	155.7	0.147
5	3D model with excess terms	Yes	Yes	27	0.93	171.7	0.174

0: MD～Sex+EHV+EHC

1: MD～Sex+Vv+Cv+Vi+Vr+Cr+VCr+Vr2+VCr2

2: MD～Sex+Vv+Cv+Vi+Vr+Cr+VCr+Vr2+VCr2+Vr:Cr

3: MD～Sex+Vv+Cv+Sv+Vi+Si+VCi+Vh+Vr+Cr+VCr+Ir+Vr2+VCr2

4: MD～Sex+Vv+Cv+Sv+Vi+Si+VCi+Vh+Vr+Cr+VCr+Ir+Vr2+VCr2+Vr:Cr+Sr:VCr+VCr:Ir

5: MD～Sex+Vv+Cv+Sv+Vi+Ci+Si+VCi+Ii+Vh+Ch+Sh+Vr+Cr+VCr+Ir+Vr2+Cr2+Sr2+VCr2+Ir2+Vr:Cr+Cr:Sr+Sr:Vr+VCr:(Sr+Ir)

Among 3D models, incorporation of terms regarding the SCCs or interaction among the cochleae, vestibules, and SCCs resulted in an increase in the AUC value and decrease in the AIC and mean square error. The values of the AIC and mean square error were minimized for the selected 3D model (model no. 4), and the values increased for a 3D model with excess explanatory terms (model no. 5). By applying the function 'stepAIC' in the R package of 'MASS' to a 3D model (model no. 5), the selected 3D model remained (model no. 4), signifying that the selected model was the optimized one. The results shown in the table also imply that improvement in MD diagnostic accuracy of the selected 3D model from the 2D model was achieved by incorporating terms related to the SCCs and interaction between the cochleae, vestibules, and SCCs, although the EV volume ratio in the SCCs was not a significant factor for MD in the analysis.
